# PD-L1 expression and its association with clinicopathological and computed tomography features in surgically resected non-small cell lung cancer: a retrospective cohort study

**DOI:** 10.1038/s41598-025-10437-9

**Published:** 2025-07-07

**Authors:** Shiwei Wang, Mingyue Xu, You Liu, Xiaoming Hou, Zhaofeng Gao, Jingjie Sun, Leilei Shen

**Affiliations:** 1 Department of Interventional Radiology, Hainan Hospital of PLA General Hospital, Sanya, 572013 China; 2 Department of General Surgery, Hainan Hospital of PLA General Hospital, Sanya, 572013 China; 3 Department of Pathology, Hainan Hospital of PLA General Hospital, Sanya, 572013 China; 4 Department of Radiology, First Medical Center of PLA General Hospital, Beijing, 100853 China; 5 School of Medicine, Nankai University, Tianjin, 300110 China; 6 Department of Oncology, Hainan Hospital of PLA General Hospital, Sanya, 572013 China; 7 Department of Thoracic Surgery, Hainan Hospital of PLA General Hospital, Jiang-Lin Road, Hai Tang District, Sanya, 572013 China

**Keywords:** Programmed death-ligand 1, Non-small cell lung cancer, Clinicopathology, Genomic mutation, Computed tomography, Surgical oncology, Cancer imaging

## Abstract

Although some studies have assessed the correlation between clinicopathological features and programmed death-ligand 1 (PD-L1) in patients with non-smallcell lung cancer (NSCLC), few studies have focused on computed tomography (CT) signs. The results of some previous studies are inconsistent and contradictory. Therefore, this study aimed to analyze the clinicopathological and CT features of NSCLC patients with different PD-L1 expression levels. This retrospective analysis included 911 NSCLC patients who undergone pulmonary resection, and most of them were early-stage. PD-L1 expression was assessed by immunohistochemistry with the Dako PD-L1 22C3 pharmDx kit. Clinicopathological features and CT signs were investigated according to different PD-L1 expression levels. The prevalence of PD-L1 expression in the resected NSCLC patients was 46.9% (427/911), with 381 patients low expression (41.8%) and 46 patients high expression (5%). Male sex, current smoking status, higher positron emission tomography-computed tomography (PET-CT) SUVmax, and elevated serum carcinoembryonic antigen levels were more frequently observed in patients with high PD-L1 expression. The frequencies of squamous cell carcinoma type, spread through air space (STAS), *TP53* mutations, advanced pathological stages, and micropapillary/solid subtype were significantly higher in patients with PD-L1 positive tumors (TPS ≥ 1%) than in those with PD-L1 negative tumors (TPS < 1%). *ALK* rearrangement was higher in patients with low-expression, and PD-L1-positive patients had bigger tumor diameter. The proportion of solid nodules and consolidation to tumor were higher in high-expression than those in the low- or negative expression PD-L1 patients. Regarding structural characteristic features, there were no differences in the frequency of irregular borders, pleural retraction, air bronchograms, bubble-like lucency or cavities, and vascular convergence among the three groups. The frequency of lobulated margins and spiculation was significantly higher in patients with PD-L1 positive tumors than in those with PD-L1 negative tumors. Patients with PD-L1 expression in NSCLC often exhibit certain clinicopathological characteristics. CT features may not reliably correlate with PD-L1 expression across different stages of lung cancer. Immunohistochemistry (IHC), using relevant kits, remains essential for further evaluation of PD-L1 expression levels.

## Introdcution

Lung cancer stands as the foremost cause of cancer-related deaths globally, with non-small cell lung cancer (NSCLC) constituting approximately 85% of all cases^1^. In recent years, the advent of checkpoint blockade immunotherapy has profoundly transformed the treatment paradigm for lung cancer, particularly through the utilization of monoclonal antibodies targeting programmed death-1/programmed death-ligand 1 (PD-1/PD-L1)^2–5^. Currently, PD-L1 expression stands as the sole validated predictive biomarker for PD-1/PD-L1 inhibitors in NSCLC patients, emphasizing the need for precise patient selection to maximize therapeutic benefits. The 22C3 anti-PD-L1 antibody has emerged as the most extensively employed diagnostic tool^6^.

Prior investigations have explored the relationship between PD-L1 expression and clinicopathological as well as genomic mutation status, yielding disparate findings^7–12^. Some studies have indicated that factors such as smoking history, disease stage, and squamous cell carcinoma (SCC) may be associated with heightened PD-L1 expression. Moreover, mutations in *epidermal growth factor receptor* (*EGFR*), *anaplastic lymphoma kinase* (*ALK*), *kirsten rat sarcoma viral oncogene homolog* (*KRAS*), and *TP53* have been linked to varying levels of PD-L1 expression^8–11^. Nevertheless, scant attention has been devoted to identifying radiological features associated with PD-L1 expression in surgically resected NSCLC.

Thus, this study aims to retrospectively examine the clinicopathological features and computed tomography signs corresponding to different PD-L1 expression levels in NSCLC patients. The insights garnered from our investigation are poised to enhance our comprehension of PD-L1 expression from a radiological standpoint.

## Methods

### Study population

**Fig. 1:**
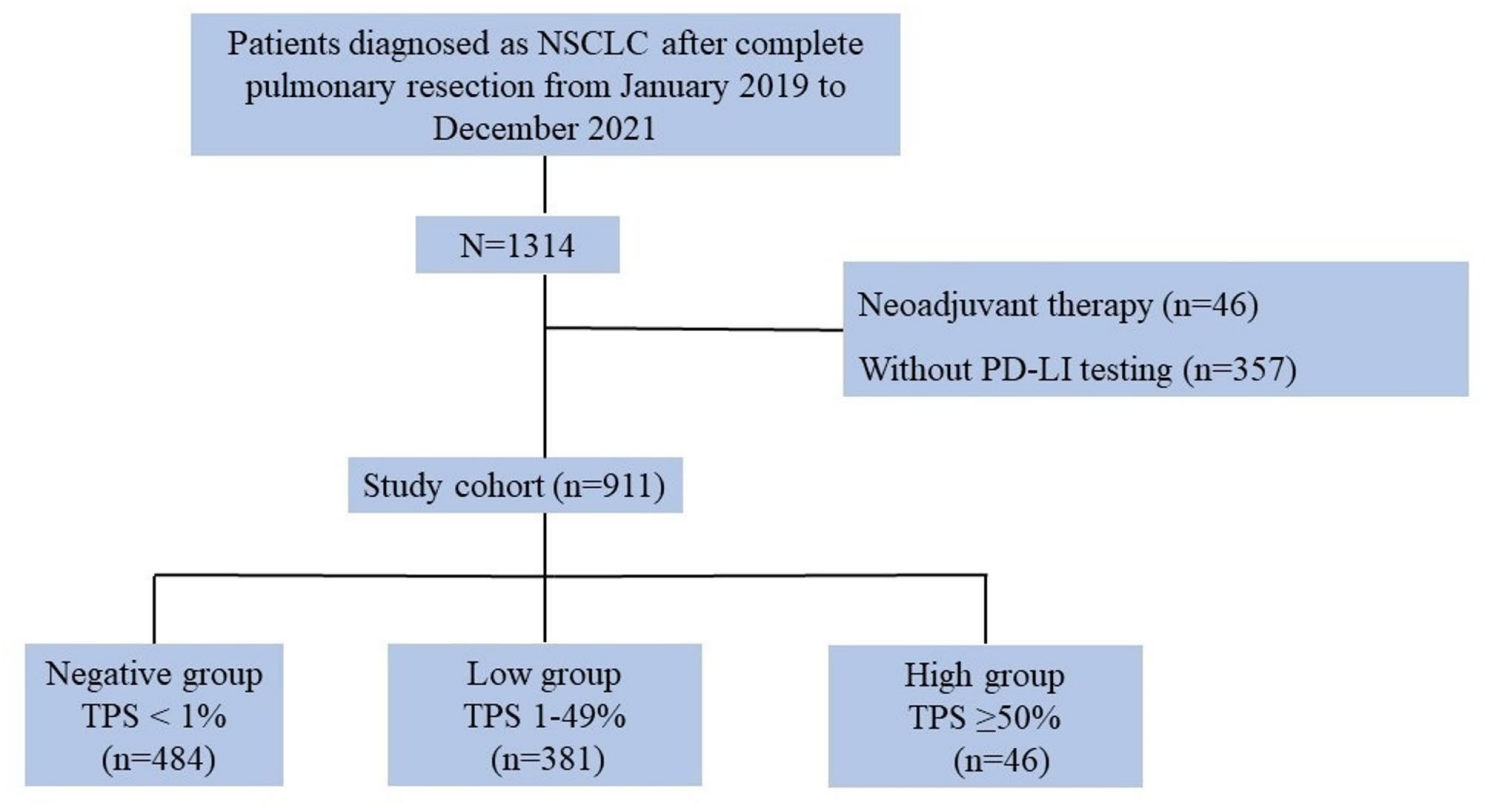
Patients flow diagram.

A total of 911 NSCLC patients’ surgically resected tissue samples were collected between January 2019 and December 2021 at the Chinese People’s Liberation Army General Hospital. Patients were included if they met the following criteria: (a) NSCLC with PD-L1 testing on specimens, (b) preoperative thin-section CT scan performed < 3 months before surgery. Patients who received neoadjuvant chemotherapy, immunotherapy, targeted therapy, or radiotherapy, or had specimen slides with less than 100 viable tumor cells or a percentage of tumor cells < 10%, were excluded (Fig. 1). Data on age, sex, smoking status, family history of lung cancer, preoperative serum carcinoembryonic antigen (CEA) level, histological subtype, and pathological TNM stage were collected for each patient. Tumor histology was classified using the World Health Organization (WHO) criteria^13^. The pathological stage was determined by the 8th TNM staging manual^14^.

### Immunohistochemical evaluation of PD-L1 expression

**Fig. 2:**
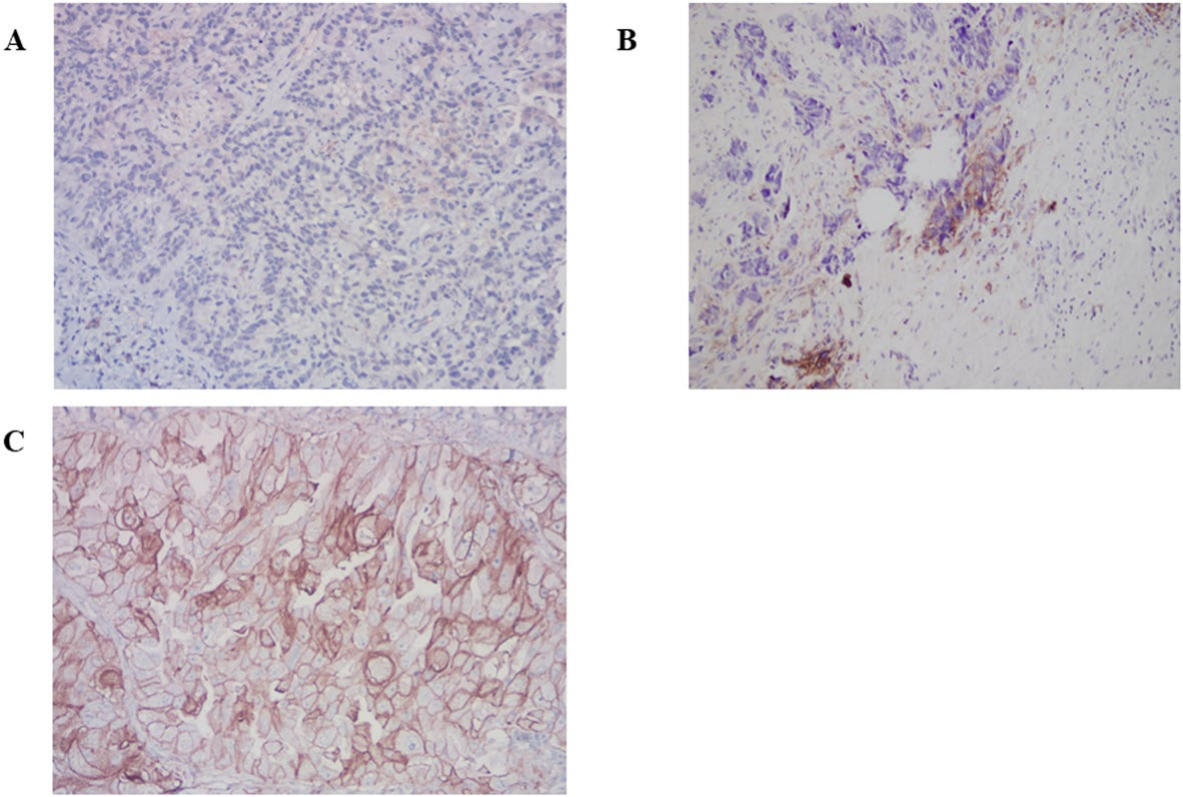
Representative IHC images of PD-L1 expression in NSCLC. (A) Negative staining for PD-L1 (TPS < 1%), (B) weakly positive membrane staining for PD-L1 (TPS 1–49%), and (C) strongly positive membrane staining for PD-L1 (TPS ≥ 50%).

Genecast Technology (Wuxi, China) conducted the entire NGS process, following the manufacturer’s scheme. PD-L1 immunohistochemistry (IHC) staining was performed using monoclonal mouse antihuman PD-L1 antibody (clone 22C3, *n* = 750, Dako: Cat No. M3653). PD-L1 positivity was defined based on the proportion of stained tumor cells (tumor proportion score [TPS]) on the TMA, according to a clinical trial assay that maximally predicts the clinical response of patients with NSCLC treated with pembrolizumab^15^. PD-L1 expression levels were classified based on the TPS as negative (< 1%) (Fig. 2A), low expression (1–49%) (Fig. 2B), and high expression (≥ 50%) (Fig. 2C).

### CT imaging and characteristics

**Fig. 3:**
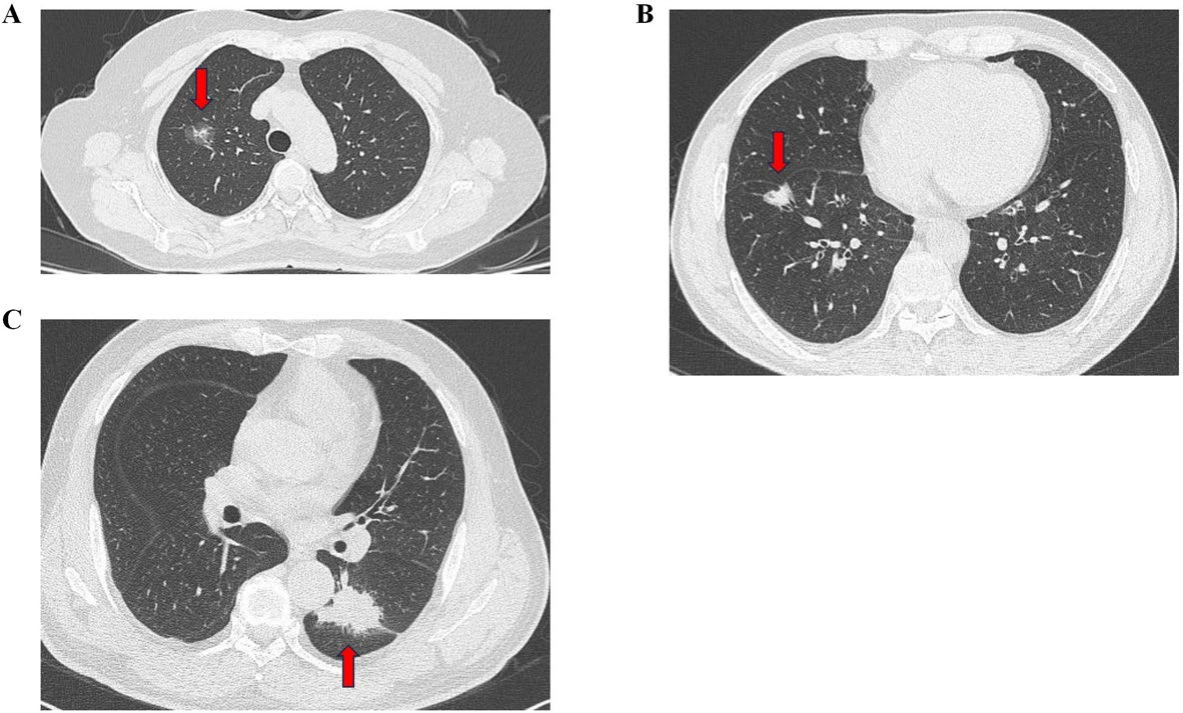
Representative CT images of PD-L1 expression in NSCLC. (A) Negative, (B) TPS 1–49%, and (C) TPS ≥ 50%.

Chest CT examinations were conducted using a Discovery CT750 HD scanner (GE Healthcare, Milwaukee, WI, USA) with the following parameters: 120 kVp, 150–200 mA, and 1.25 mm reconstruction thickness with a 1.25 mm reconstruction interval. Two radiologists (S. W. Wang and X. M. Hou, with 5 and 10 years of experience in chest CT diagnosis, respectively) independently reviewed the images. They were informed that all patients had NSCLC but were blinded to the PD-L1 expression status (Fig. 3). The CT characteristics assessed included tumor size, tumor density (solid/ground-glass opacity), consolidation tumor ratio (CTR = consolidation size/tumor size), lesion location (central or peripheral), round or irregular shape, lobulated or spiculated margins, presence of air bronchograms, bubble-like lucency or cavities, and pleural retraction. Ground-glass opacity (GGO) encompasses pure GGO and mixed GGO was evaluated by the CTR.

### Statistical analysis

All statistical analyses were performed using SPSS version 22 software (IBM Corporation, Armonk, NY, USA). Every group with categorical variables for patient demographics and clinical characteristics was summarized using the frequency and percentage, and statistical comparisons between the three PD-L1 expression category groups were performed using the Pearson’s χ^2^ or Fisher exact tests. Bonferroni-adjusted pairwise comparisons were employed to control Type I error inflation. Continuous variables were expressed as means with standard deviations, as well as medians with a range of values. The analysis protocol began with omnibus tests assessing normality and homogeneity of variance. When the assumption of equal variances was met, we performed post-hoc comparisons with the least significant difference (LSD) method; when this assumption was violated, Tamhane’s T2 test was applied instead. For non-parametric data, we employed the Kruskal-Wallis one-way ANOVA, in which the Bonferroni correction adjusted significance levels for multiple comparisons, minimizing the probability of Type I statistical errors. The significance threshold for the overall test was set at *P* < 0.05. Only when the overall test showed significant results (*P* < 0.05) were subsequent pairwise comparisons performed. As this study involved three groups, requiring three pairwise comparisons, the Bonferroni correction was applied to control for Type I error, thereby establishing *P* < 0.0167 as the final criterion for statistical significance.

## Results

### Patients’ baseline characteristics

PD-L1 expression was negative in 484 (53.2%) of 911 patients, low in 381 (41.8%), and high in 46 (5%). The clinical characteristics are summarized in Table 1. Male sex, current smoking status, higher positron emission tomography-computed tomography (PET-CT) SUVmax, and elevated serum carcinoembryonic antigen levels were more frequently observed in patients with high PD-L1 expression. There were no differences among the three groups regarding age and family history of lung cancer.

**Table 1:** Patient’s baseline characteristics.

Variables	PD-L1 expression (TPS)			*P*-value
	Negative, < 1% (*n* = 484, 53.2%)	Low, 1–49% (*n* = 381, 41.8%)	High, ≥ 50% (*n* = 46, 5.0%)	
Age (years)	58.96 ± 9.33(23–79)	59.33 ± 9.12(31–80)	61.41 ± 6.81(46–75)	0.214
≥ 65 y	347 (71.7)	264 (69.7)	30 (65.2)	0.547
< 65 y	137 (28.3)	117 (30.7)	16 (34.8)	0.547
Sex				0.001^a^
Male	208 (43) *#	198 (52)	31 (67.4)	
Female	276 (57) *#	183 (48)	15 (32.6)	
Smoking status				< 0.001^b^
Nonsmoker	361 (74.6) *#	253 (66.4)	23 (50)	
Smoker	123 (25.4) *#	128 (33.6)	23 (50)	
Family history of lung cancer	75 (15.5)	50 (13.1)	5 (10.9)	0.487
PET-CT	3.4 (1.73, 7.25)	3.7 (1.95, 6.95) #	9.50 (5.45, 11.83)	< 0.001^c^
CEA level				0.016^d^
Normal (0–5 ng/mL)	449 (92.8) #	347 (91.1)	37 (80.4)	
Abnormal (> 5 ng/mL)	35 (7.2)	34 (8.9)	9 (19.6)	

Note: Unless otherwise indicated, data are shown as numbers with percentages in parentheses. * *P* < 0.0167 vs. low group; # *P* < 0.0167 vs. high group.

PD-L1, programmed death-ligand 1; TPS, tumor proportion score; PET-CT, positron emission tomography-computed tomography; CEA, carcinoembryonic antigen.

^a^ Pearson χ^2^ test: Negative vs. Low: *P* = 0.009; Negative vs. High: *P* = 0.001; Low vs. High: *P* = 0.048.

^b^ Pearson χ^2^ test: Negative vs. Low: *P* = 0.008; Negative vs. High: *P* < 0.001; Low vs. High: *P* = 0.028.

^c^ Kruskal-Wallis test: Negative vs. Low: *P* = 1.000; Negative vs. High: *P* < 0.001; Low vs. High: *P* < 0.001.

^d^ Pearson χ^2^ test: Negative vs. Low: *P* = 0.362; Negative vs. High: *P* = 0.004; Low vs. High: *P* = 0.035.

### Correlations between PD-L1 expression and pathological and molecular characteristics

Comparisons of pathological findings and molecular characteristics are presented in Table 2. The frequency of squamous cell carcinoma type, spread through air space (STAS), *TP53* mutations, advanced pathological stages, and lymph node metastasis was significantly higher in patients with PD-L1-positive tumors (TPS ≥ 1%) than in those with PD-L1-negative tumors (TPS < 1%). Poor cell differentiation and the micropapillary/solid subtype were more frequently observed in patients with high PD-L1 expression than in those with low or negative expression. The Ki-67 index was higher in patients with strong PD-L1 expression than in those with weak or negative expression. However, there were no differences among the three groups regarding to mucinous type, visceral pleural invasion (VPI), lymphovascular invasion, tumors with *EGFR* mutation, tumors with *KRAS* mutation, tumors with *BRAF-V600E* mutation, or tumors with *ROS1* mutation. *ALK* rearrangement was higher in patients with low expression, and PD-L1-positive patients had a larger tumor diameter than those with PD-L1-negative tumors.

**Table 2:** Pathological and molecular characteristics.

Variables	PD-L1 expression (TPS)			*P*-value
	Negative, < 1% (*n* = 484, 53.2%)	Low, 1–49% (*n* = 381, 41.8%)	High, ≥ 50% (*n* = 46, 5.0%)	
Pathological type				< 0.001^a^
SCC	7 (1.4) *#	36 (9.4)	8 (17.4)	
ADC	473 (97.7) *#	342 (89.8)	38 (82.6)	
Others	4 (0.8) *#	3 (0.8)	0	
Tumor diameter	20 (15, 25) #	20 (15, 25)	30 (19.3, 32.8)	0.002^b^
Cell differentiation				< 0.001^c^
Well	99 (20.5) *#	28 (7.3) #	0	
Moderate	287 (59.3) *#	221 (58) #	17 (37)	
Poor	98 (20.2) *#	132 (34.6) #	29 (63)	
Mucinous	40 (8.3)	38 (10)	6 (13)	0.452
Micropapillary/solid subtype	108 (22.3) *#	142 (37.3) #	28 (60.9)	< 0.001^d^
VPI	206 (42.6)	195 (51.2)	22 (47.8)	0.051
Lymphovascular invasion	12 (2.5)	21(5.5)	2 (4.3)	0.069
STAS	13 (2.7) *#	29 (7.6)	5 (10.9)	0.001^e^
Ki-67 (IQR)	7.5 (5, 15) *#	20 (10, 40) #	60 (47.5, 60)	< 0.001^f^
*EGFR*	159/228 (69.7)	85/135 (63)	10/17 (58.8)	0.321
*ALK*	36/483 (7.5) *	51/375 (13.6)	3/46 (6.5)	0.009^g^
*KRAS*	14/218 (6.4)	14/130 (10.8)	3/16 (18.8)	0.121
*TP53*	31/188(16.5) *#	39/101(38.6)	8/13 (61.5)	< 0.001^h^
*BRAF-V600E*	3/218 (1.4)	3/129 (2.3)	0	0.694
*ROS1*	1/217 (0.5)	3/129 (2.3)	1/16 (6.3)	0.083
LND stations	5 (5, 6)	5 (4, 6)	6 (4.75, 6.25)	0.374
LND numbers	10 (8, 16) *#	9 (7, 14.5)	15 (11.75, 16.5)	0.001^i^
Node stage				0.002^j^
N0	456 (94.2) *#	332 (87.1)	39 (84.8)	
N1	7 (1.4) *#	21 (5.5)	2 (4.3)	
N2	21 (4.3) *#	28 (7.3)	5 (10.9)	
pTNM stage				0.001^k^
I	436 (90.1) *#	315 (82.7)	35 (76.1)	
II	25 (5.2) *#	35 (9.2)	4 (8.7)	
III	21 (4.3) *#	25 (6.6)	4 (8.7)	
IV	2 (0.4) *#	6 (1.6)	3 (6.5)	

Note: Unless otherwise indicated, data are shown as numbers with percentages in parentheses. * *P* < 0.0167 vs. low group; # *P* < 0.0167 vs. high group.

SCC, squamous cell carcinoma; ADC, adenocarcinoma; VPI, visceral pleural invasion; STAS, spread through air space; IQR, interquartile range; PD-L1, programmed death ligand-1; *EGFR*, *epidermal growth factor receptor*; *KRAS*, *Kirsten rat sarcoma*; *TP53*, *tumor protein 53*; LND, lymph node dissection.

^a^ Kruskal-Wallis test: Negative vs. Low: *P* < 0.001; Negative vs. High: *P* < 0.001; Low vs. High: *P* = 0.194.

^b^ Kruskal-Wallis test: Negative vs. Low: *P* = 0.022; Negative vs. High: *P* = 0.016; Low vs. High: *P* = 0.340.

^c^ Kruskal-Wallis test: Negative vs. Low: *P* < 0.001; Negative vs. High: *P* < 0.001; Low vs. High: *P* = 0.001.

^d^ Pearson χ^2^ test: Negative vs. Low: *P* < 0.001; Negative vs. High: *P* < 0.001; Low vs. High: *P* = 0.002.

^e^ Pearson χ^2^ test: Negative vs. Low: *P* = 0.001; Negative vs. High: *P* = 0.003; Low vs. High: *P* = 0.394.

^f^ Kruskal-Wallis test: Negative vs. Low: *P* < 0.001; Negative vs. High: *P* < 0.001; Low vs. High: *P* < 0.001.

^g^ Pearson χ^2^ test: Negative vs. Low: *P* = 0.003; Negative vs. High: *P* = 1.000; Low vs. High: *P* = 0.175.

^h^ Pearson χ^2^ test: Negative vs. Low: *P* < 0.001; Negative vs. High: *P* = 0.001; Low vs. High: *P* = 0.114.

^i^ Kruskal-Wallis test: Negative vs. Low: *P* = 0.014; Negative vs. High: *P* = 0.014; Low vs. High: *P* = 0.358.

^j^ Kruskal-Wallis test: Negative vs. Low: *P* = 0.010; Negative vs. High: *P* = 0.001; Low vs. High: *P* = 0.460.

^k^ Kruskal-Wallis test: Negative vs. Low: *P* = 0.005; Negative vs. High: *P* = 0.016; Low vs. High: *P* = 0.515.

### PD-L1 expression and visual CT signs

The findings related to visual CT signs based on different PD-L1 expression levels are summarized in Table 3. No significant differences were observed in tumor location or anatomical type among the three groups. However, tumor size was notably smaller in PD-L1-negative patients compared to those with low or high PD-L1 expression. The proportion of solid nodules was higher in patients with high PD-L1 expression than in those with low or negative expression, while the consolidation tumor ratio (CTR) was lowest in the PD-L1-negative group. In terms of structural characteristics, there were no notable differences in the frequency of irregular borders, pleural retraction, air bronchograms, bubble-like lucency or cavities, and vascular convergence among the three groups. However, the frequency of lobulated margins and spiculation was significantly higher in patients with PD-L1-positive tumors (TPS ≥ 1%) compared to those with PD-L1-negative tumors (TPS < 1%).

**Table 3:** CT signs in different PD-L1 expression groups.

Variables	PD-L1 expression (TPS)			*P*-value
	Negative, < 1% (*n* = 484, 53.2%)	Low, 1–49% (*n* = 381, 41.8%)	High, ≥ 50% (*n* = 46, 5.0%)	
Tumor size, mm (IQR)	21 (15.5, 28) *#	27 (18, 35.5)	28.5 (21.3, 43.5)	< 0.001^a^
Tumor location				0.659
Left upper lobe	130 (26.9)	93 (24.4)	12 (26.1)	
Left lower lobe	68 (14)	59 (15.5)	11 (23.9)	
Right upper lobe	162 (33.5)	118 (31)	13 (28.3)	
Right middle lobe	37 (7.6)	38 (10.0)	3 (6.5)	
Right lower lobe	87 (18)	73 (19.2)	7 (15.2)	
Anatomical type				0.114
Central	14 (2.9)	16 (4.2)	4 (8.7)	
Peripheral	474 (97.1)	365 (95.8)	42 (91.3)	
CT density				
Solid nodule	172 (35.5) *#	239 (62.7) #	43 (93.5)	< 0.001^b^
GGO	312 (64.5) *#	142 (37.3) #	3 (6.5)	< 0.001^b^
CTR	0.76 (0.40, 1.00) *#	1 (0.75, 1.00) #	1.00 (1.00, 1.00)	< 0.001^c^
Irregular	387 (80)	316 (82.9)	41 (5.5)	0.216
Lobulated	375 (77.5) *#	322 (84.5)	43 (93.5)	0.003^d^
Spiculation	306 (63.2) *	276 (72.4)	37 (80.4)	0.003^e^
Bubble-like lucency or cavities	219 (45.2)	146 (38.3)	13 (28.3)	0.021^f^
Pleural retraction	359 (74.2)	288 (75.6)	36 (78.3)	0.776
Vascular convergence	403 (83.3)	339 (89)	43 (93.5)	0.018^g^
Air bronchogram	197 (40.7)	176 (46.2)	24 (52.2)	0.131

Note: Unless otherwise indicated, data are shown as numbers with percentages in parentheses. * *P* < 0.0167 vs. low group; # *P* < 0.0167 vs. high group.

IQR, interquartile range; CT, computed tomography; GGO, ground-glass opacity; CTR, consolidation tumor ratio.

^a^ Kruskal-Wallis test: Negative vs. Low: *P* < 0.001; Negative vs. High: *P* < 0.001; Low vs. High: *P* = 0.126.

^b^ Pearson χ^2^ test: Negative vs. Low: *P* < 0.001; Negative vs. High: *P* < 0.001; Low vs. High: *P* < 0.001.

^c^ Kruskal-Wallis test: Negative vs. Low: *P* < 0.001; Negative vs. High: *P* < 0.001; Low vs. High: *P* = 0.001.

^d^ Pearson χ^2^ test: Negative vs. Low: *P* = 0.009; Negative vs. High: *P* = 0.011; Low vs. High: *P* = 0.103.

^e^ Pearson χ^2^ test: Negative vs. Low: *P* = 0.004; Negative vs. High: *P* = 0.020; Low vs. High: *P* = 0.247.

^f^ Pearson χ^2^ test: Negative vs. Low: *P* = 0.041; Negative vs. High: *P* = 0.026; Low vs. High: *P* = 0.183.

^g^ Pearson χ^2^ test: Negative vs. Low: *P* = 0.017; Negative vs. High: *P* = 0.070; Low vs. High: *P* = 0.452.

## Discussion

In this study, we explored the clinicopathological and radiological characteristics of NSCLC patients with varying PD-L1 expression levels. Our findings revealed several key observations: (I) Male sex, current smoking status, higher PET-CT SUVmax, and elevated CEA level were more prevalent in patients with stronger PD-L1 expression; (II) Patients with PD-L1 positive tumors (TPS ≥ 1%) showed a higher frequency of squamous cell carcinoma type, STAS, *TP53* mutations, advanced pathological stages, lymph node metastasis, poor cell differentiation, and micropapillary/solid subtype in ADC. However, no differences were observed regarding *EGFR* mutation, *KRAS* mutation, *BRAF-V600E* mutation, or *ROS1* mutation; (III) radiologically, tumor size and CTR were significantly smaller in PD-L1-negative patients. CT features were not predictive of PD-L1 expression.

Previous studies have reported that patients with increased PD-L1 expression exhibit certain clinicopathological or molecular features in lung cancer^7–12^. Consistent with these reports, we observed that male sex, current smoking status, higher PET-CT SUVmax, and elevated CEA level were more common in patients with higher PD-L1 expression^8–10,16,17^. Pathologically, PD-L1 expression varied among different subtypes, with high expression more frequently observed in SCC patients and in the solid or micropapillary predominately ADC subtype^12,16,17^. Poorly differentiated advanced stage cases also showed a greater tendency for stronger PD-L1 expression^9^. Additionally, our study found that larger diameter tumors and higher Ki-67 index were associated with higher PD-L1 expression. STAS was considered as a novel inform of invasion by 2015 WHO classifications of lung tumors^18^ and was recommended to introduce as a histologic descriptor in the 9th edition of the TNM manual^19^. Our study representd that the prevalence of STAS was significantly higher in patients with PD-L1 positive tumors (TPS ≥ 1%) than in those with PD-L1 negative tumors (TPS < 1%), which was the first report, to the best of our knowledge. Nonetheless, we failed to find similar results regarding the LVI and VPI. From these we can see that the expression of PD-L1 was associated with the progress of cancer. Higher PET-CT SUVmax, elevated CEA level, micropapillary or solid subtype, poor cell differentiation, bigger diameter, more STAS, and higher Ki-67 index all indicated the advanced stage of tumors with a higher tumor mutation burden, whether node stage or pTNM stage. Stronger PD-L1 expression appears to correlate with more aggressive tumors.

Previous studies have examined the correlation between PD-L1 expression and prognosis, revealing that this correlation can vary depending on driver oncogene mutations^7^. However, the genetic results seemed to be inconsistent. Some studies have suggested a link between stronger PD-L1 expression and *EGFR* wild-type tumors, but not with *KRAS* alterations or *ALK* rearrangements^7,20^. In our study, we found that PD-L1 expression was increased in *TP53* mutated and *ALK* fusion tumors, but did not correlate with *EGFR*, *KRAS*, *BRAF-V600E*, or *ROS1* mutation status (*P > 0.05*). Positive PD-L1 expression was observed in approximately 62.5% (95/152) of *EGFR*-mutated NSCLC patients, which was higher than in other studies^16,21^. Interestingly, *ALK*-rearranged tumors had a significantly higher proportion of PD-L1 expression compared to wild-type patients (12.8% vs. 7.5%) in our study, contradicting other reports^16,17,22^. Some studies also revealed that positive association between PD-L1 expression and *KRAS* or *TP53* mutations, which our study supports^23,24^. However, the relationship between PD-L1 expression and genetic status is complex and may not reach a definitive conclusion in the future. Intratumor heterogeneity may contribute to this complexity, as different areas of a tumor can have diverse results. For example, in adenocarcinoma (ADC) patients, lepidic-predominant lesions may have negative or low PD-L1 expression but are more likely to harbor *EGFR* mutations. Conversely, high PD-L1 expression is more frequently observed in solid or micropapillary predominant ADC patients without genetic *EGFR* or *ALK* alterations. This complexity is primarily influenced by the tumor’s pathological stage. Currently, it is generally believed that anti-PD-1/PD-L1 therapy may not benefit *EGFR*- or *ALK* fusion- mutated NSCLC patients and could even lead to severe adverse effects^21,22,25^. However, there are increasing reports of patients turning to immunotherapy after developing resistance to *EGFR*- or *ALK*-TKIs^26^. Therefore, with a thorough understanding of gene mutations and the PD-1/PD-L1 axis, it may be possible to find a reasonable solution to this seemingly incompatible relationship.

Radiomics and deep learning techniques have been widely explored to extract CT-based features for predicting PD-L1 expression in NSCLC^27^. Prior studies have investigated the application of radiogenomics to PD-L1 expression^28–31^. Sun et al.‘s prediction model, which incorporated a radiomics signature and clinical variables, demonstrated potential in forecasting PD-L1 expression in NSCLC patients, achieving areas under the curve (AUC) of 0.829 and 0.848 in the training and validation cohorts, respectively^32^. Similarly, prediction models combining clinical risk factors and CT radiomic features may enable noninvasive assessment of PD-L1 expression^27,33^. Zheng XX et al.‘s combined model for PD-L1 ≥ 1%, which included a radiomics score, white blood cell counts, and air bronchogram, achieved the highest performance^34^. Our study compared CT features across different PD-L1 expression levels and found that CT characteristics do not appear to predict PD-L1 expression. Tumor size, proportions of solid nodules, and the CTR were significantly lower in the PD-L1-negative group, also indicating the progression of the tumor, such as nodal metastases, Ki-67 index, LVI, and STAS in the pathological profile. Radiologically, the frequency of irregular borders, pleural retraction, and air bronchograms were comparable between PD-L1 negative and positive tumors. Therefore, the combined model may serve as a promising method for predicting PD-L1 expression, even for assessing immunotherapy. However, we still consider IHC staining as the sole standard predictor for PD-L1, not radiomics, with or without clinical characteristics.

Our study had several limitations. First, this retrospective study, conducted at a single thoracic center, enrolled only a small number of patients with high-expression PD-L1 (46 patients). This may be due to the enrollment of early-stage patients diagnosed post-surgery rather than advanced-stage patients diagnosed by biopsy. Secondly, we focused on clinicopathological and radiological features but failed to create a prediction model for PD-L1 expression using a multicenter dataset. Third, we lacked follow-up data to evaluate disease recurrence or survival based on different PD-L1 expression levels. Fourth, interobserver variability was not thoroughly addressed when evaluating CT images. A final limitation is that our study evaluated the association between PD-L1 expression and clinical, radiological, and pathological factors solely through univariate analysis without adjusting for potential confounding effects among variables. These findings should therefore be interpreted with caution.

## Conclusions

Patients with PD-L1 expression in NSCLC often exhibit certain clinicopathological characteristics, including male sex, current smoking status, elevated PET-CT SUVmax, increased CEA levels, squamous cell carcinoma (SCC) type, spread through air spaces (STAS), *TP53* mutations, advanced pathological stages, poor cellular differentiation, and the micropapillary/solid subtype in adenocarcinoma (ADC). CT features may not reliably correlate with PD-L1 expression across different stages of lung cancer. Immunohistochemistry (IHC), using relevant kits, remains essential for further evaluation of PD-L1 expression levels.

## Data Availability

The datasets used and/or analyzed during the current study are available from the corresponding author on reasonable request.

## Abbreviations

NSCLC: Non-small cell lung cancer
ALK: Anaplastic lymphoma kinase
CEA: Carcinoembryonic antigen
CT: Computed tomography
GGO: Ground-glass opacity
CTR: Consolidation tumor ratio
IHC: Immunohistochemistry
NGS: next-generation sequencing
SCC: Squamous cell carcinoma
VPI: Visceral pleural invasion
PD-L1: Programmed death ligand-1
EGFR: Epidermal growth factor receptor
KRAS: Kirsten rat sarcoma viral oncogene
TP53: Tumor protein 53
TPS: Tumor proportion score
PET-CT: Positron emission tomography-computed tomography
STAS: Spread through air space
LND: Lymph node dissection
